# DNA mismatch repair in the context of chromatin

**DOI:** 10.1186/s13578-020-0379-7

**Published:** 2020-02-03

**Authors:** Yaping Huang, Guo-Min Li

**Affiliations:** Department of Radiation Oncology, University of Texas Southwestern Medical Center, Dallas, TX 75390 USA

## Abstract

DNA mismatch repair (MMR) maintains replication fidelity by correcting mispaired nucleotides incorporated by DNA polymerases. Defects in MMR lead to cancers characterized by microsatellite instability. Recently, chromatin mechanisms that regulate MMR have been discovered, which sheds new light on MMR deficiency and its role in tumorigenesis. This review summarizes these chromatin-level mechanisms that regulate MMR and their implications for tumor development.

## Background

DNA mismatch repair (MMR) maintains replication fidelity in a replication-coupled manner, primarily by correcting misincorporated nucleotides in the nascent DNA strand [1–4]. The typical MMR reaction in human cells comprises three major steps. First, the mismatch recognition protein MutSα (MSH2–MSH6 heterodimer) or MutSβ (MSH2–MSH3 heterodimer) recognizes the mismatch, which triggers concerted interactions with proliferating cell nuclear antigen (PCNA) and MutLα (MLH1–PMS2 heterodimer), leading to the recruitment of exonuclease 1 (EXO1) to a single-strand DNA break. Then, Exo1-catalyzed DNA excises the mispaired base from the nick up to and beyond the mismatch in a manner dependent on MutSα (or MutSβ), MutLα, and replication protein A (RPA). Finally, the DNA gap is filled by DNA polymerase δ in the presence of PCNA, RPA, and replication factor C (RFC), followed by DNA ligase I-catalyzed nick ligation.

In eukaryotes, DNA is wrapped around histone octamers, which, together with DNA, compose nucleosomes to form chromatin. Thus, all DNA metabolic reactions, including MMR, are precisely regulated by the structures of chromatin, particularly its component histone proteins and their modifications. Indeed, recent studies have indicated that trimethylation of histone H3 lysine 36 (H3K36me3) plays a role in MMR by recruiting MutSα to replicating chromatin [5]. In addition, chromatin assembly/remodeling factors also interact with MMR proteins to coordinate MMR and nucleosome formation [6, 7].

Loss-of-function mutations or promoter hyper-methylation of MMR genes, such as *MSH2* and *MLH1,* increase susceptibility to cancers, including hereditary non-polyposis colorectal cancer (HNPCC), also called lynch syndrome [1, 2, 4, 8, 9]. The demonstration of H3K36me3 as an essential component for MMR in vivo [5] may provide new insights into MMR deficiency and cancer susceptibility in the chromatin context. H3K36me3 is a well-known histone post-translational modification mark, and its cellular level is regulated by its trimethyltransferase SETD2 and lysine demethylase KDM4 [10]. Inappropriate expression of and/or defects in these histone writer and eraser genes probably influence cellular H3K36me3 levels, leading to the loss of MMR function.

Recent studies have also shown that mutations in histone 3 (H3), particularly H3K36M/I and H3G34V/R, which frequently occur in various tumors [11–14], affect H3K36me3 expression levels [15, 16]. This review will focus on the most recent developments in the field concerning the regulation of MMR in the context of chromatin and its association with cancer susceptibility. Readers are also referred to several recent reviews in this area [17–20].

## Main text

### H3K36me3 distribution dictates local mutation frequency

The biochemistry of MMR is essentially well established, but how the MMR system is regulated in the chromatin context is not fully understood. In 2013, Li et al. [5] identified H3K36me3 as an important MMR regulator in vivo by recruiting mismatch recognition protein MutSα to replicating chromatin through its physical interaction with the PWWP domain of human MutSα. A recent chromatin immunoprecipitation followed by sequencing (ChIP-Seq) study [21] has illustrated the genome-wide distribution of H3K36me3 and MutSα in HeLa cells. Both H3K36me3 and MutSα are unevenly distributed in chromatin [10, 21]. H3K36me3 and MutSα are more enriched in euchromatin, exons, and 3′ gene bodies than in heterochromatin, introns, and 5′ gene bodies, respectively. Consistent with MMR’s genome maintenance role, the H3K36me3–MutSα distribution is inversely correlated with mutation frequencies in these genomic regions, as the mutation frequencies in euchromatin, exons, and 3′ gene bodies are much lower than in heterochromatin, introns, and 5′ gene bodies, respectively. In other words, the abundance of H3K36me3 and MutSα is inversely correlated to the local mutation frequency [21].

It is known that replication timing determines mutation frequency: early replicating genes exhibit lower mutation frequencies than late replicating genes. This phenomenon can be well explained by the distribution and enrichment of H3K36me3/MutSα in chromatin. Analyzing the relationship between replication timing, H3K36me3/MutSα enrichment, and mutation frequency revealed that early replicating chromatin regions are highly enriched for H3K36me3/MutSα and display a lower mutation frequency than late replicating regions, which contain fewer H3K36me3/MutSα signals [21]. These observations suggest that H3K36me3-mediated MMR ensures the replication accuracy of early replicating genes/chromatin, where actively transcribed genes are located. In addition to safeguarding actively transcribed genes during DNA replication, H3K36me3-mediated MMR also appears to protect these genes during transcription by directly or indirectly removing DNA lesions associated with transcription [21]. However, how H3K36me3 regulates MMR in different DNA transactions, e.g., replication and transcription, remains to be investigated.

It is worth noting that not all eukaryotic cells use H3K36me3 for MutSα recruitment. For example, despite that the abundance and distribution pattern of H3K36me3 in yeast genome are similar to those in human genome [22–24], yeast MSH6 does not have a PWWP domain. Thus, yeast MutSα must be recruited by a histone mark different from H3K36me3, which is consistent with the report that set2 deficiency doesn’t influence mutation frequency in yeast [25]. A recent study revealed that the PWWP domain of MSH6 in *Capsella rubella* interacts better with H3K4me3 than with H3K36me3 [26], suggesting that H3K4me3, rather than H3K36me3, is the preferred histone mark for loading MutSα to chromatin in plants. The difference in MutSα recruitment among different organisms may suggest an adapted evolution for MMR regulation in the chromatin context, although the canonical MMR function is highly conserved. Therefore, additional studies are needed to determine why different systems use different mechanisms for MutSα recruitment.

### Chromatin remodeling in MMR

**Fig. 1:**
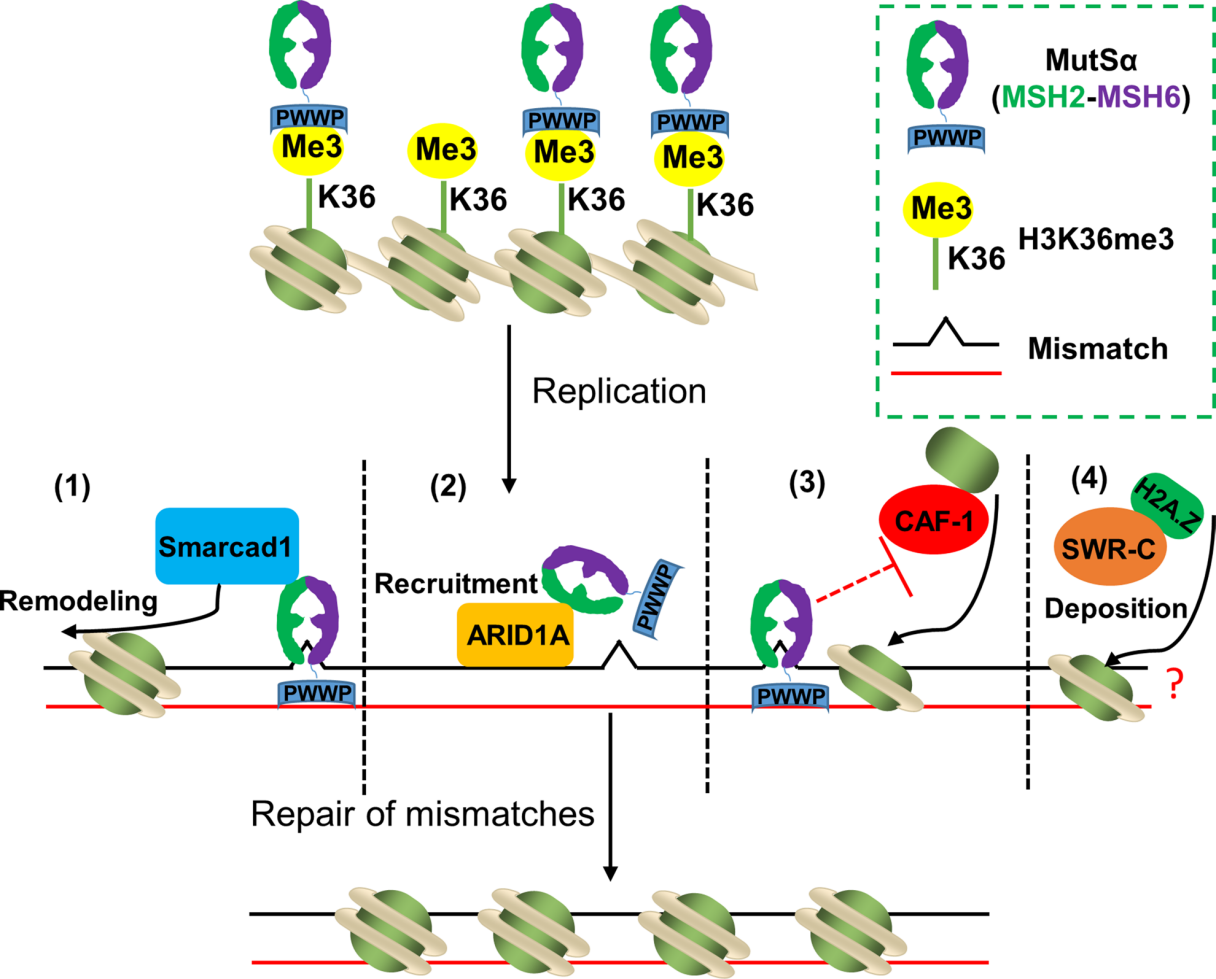
Chromatin remodeling in MMR. MMR proteins, especially MutSα, coordinate with chromatin remodelers and nucleosome assembly factors to ensure the repair of mismatches before they are packaged into nucleosomes. The current understanding of how chromatin remodeling functions in MMR is as follows: (1) at the replication fork, the disruption of nucleosomes allows MutSα to bind to DNA and search for replication-generated mismatches, and the MSH2 subunit of MutSα interacts with the chromatin remodeler Smarcad1 to exclude nucleosomes from the repair site; (2) ARID1A, a subunit of the chromatin remodeling complex SWI/SNF, interacts with MutSα through the MSH2 subunit; (3) MSH6 interacts directly with CAF-1 to inhibit CAF-1-mediated nucleosome assembly before mispaired nucleotides are corrected; (4) chromatin remodeling enzyme SWR-C-dependent H2A.Z deposition enhances mismatch repair through an unknown mechanism

MMR occurs in the chromatin context, where nucleosome obstacles must be handled for efficient repair before the newly synthesized mismatch-containing DNA is packaged into nucleosomes. Thus, MMR needs to coordinate with chromatin remodeling factors and/or nucleosome assembly factors to repair misincorporated nucleotides (Fig. 1). CAF-1 is the most studied nucleosome assembly factor involved in MMR during replication. CAF-1 directly interacts with MMR components MutSα and PCNA, and MutSα inhibits CAF-1-mediated chromatin assembly [27]. Recent biochemical studies further indicate that MutSα inhibits CAF-1- and ASF1A-H3-H4-dependent packaging of DNA mismatches into (H3–H4)_2_ tetramers [28]. These studies support the idea that MMR occurs before the mismatch is packaged into nucleosomes. In addition, CAF-1- and ASF1A-H3-H4-dependent nucleosome assembly quickly represses the unnecessary degradation of the discontinuous strand [7, 28]. Thus, nucleosome assembly is compatible with MMR during replication. In contrast to CAF-1, the chromatin remodeler Smarcad1 is recruited by MSH2 to mismatch-containing DNA to exclude nucleosomes from the repair site [29]. It is interesting to note that, although MSH2, a subunit of MutSα, can be recruited to chromatin via H3K36me3 [5], a recent study showed that ARID1A, a subunit of the chromatin remodeling complex SWI/SNF, recruits MSH2 to the chromatin during replication through direct interaction [30]. Histone variants also play important roles in DNA transactions, in addition to nucleosome assembly and chromatin remodeling. It was reported that deposition of H2A.Z by chromatin remodeling enzyme SWR-C stimulates Exo1 activity and enhances MMR during replication, although the mechanism is unclear [31]. Thus, it is worth examining how the chromatin remodeling machinery and histone variants modulate MMR in future studies.

### Histone mutations affecting H3K36me3 levels impair mismatch repair

In the past few years, driver mutation hotspots targeted to histone H3.3—including H3.3K27M, H3.3G34V/R and H3.3K36M/I amino acid substitutions—have been identified in various tumors (Table 1) [11–14, 32, 33]. The H3.3K27M and H3.3G34V/R mutation signatures are specific to pediatric glioblastomas (GBMs), making these mutation signatures new biomarkers for GBM subtyping and diagnosis. Further studies have demonstrated that pediatric GBMs can be further subtyped by K27M and G34V/R mutations [14, 34]. Functional studies have demonstrated that H3K27M is a gain-of-function mutation that results in genome-wide depletion of H3K27me3 and disrupts gene expression patterns, which promotes tumorigenesis [11, 35]. H3K36M/I substitution has been reported to block the methyltransferase from methylating H3K36, which reprograms the genome-wide H3K36 methylation pattern [12, 32]. The H3K36me3 level is dramatically lower in cells with H3K36M/I mutations [12, 33], which impairs the H3K36me3-dependent pathways, including MMR. However, until recently, the function of G34V/R remained obscure, although it was postulated that these mutations drive tumorigenesis by upregulating MYCN [34].

**Table 1:** Factors modulating H3K36me3 and their susceptibility to cancers

Function	Enzyme	Related cancers
Methyltransferase	ASH1L	Sinonasal neuroendocrine tumors, lung cancer, prostate cancer
	NSD1	Acute myeloid leukemia, prostate, neuroblastoma, breast, lung, glioma
	NSD2	Multiple myeloma, acute lymphocytic leukemia, prostate
	NSD3	Acute myeloid leukemia, breast cancer
	PRDM9	Acute lymphocytic leukemia
	SETD2	Renal clear cell carcinoma, lymphoblastic leukemia, breast cancer, prostate cancer, lung cancer, glioma, thymic carcinoma, acute myeloid leukemia
	SETD3	Renal cell carcinoma, B-cell lymphomas
	SETMAR	Acute myeloid leukemia, breast cancer
	SMYD2	Renal cell, acute myeloid leukemia, chronic lymphocytic leukemia, breast cancer
Demethylase	KDM2A	Gastric, breast, lung
	KDM2B	Pancreatic, hematologic
	KDM4A	Breast, prostate
	KDM4B	Breast, prostate, colon, gastric, lung, melanoma
	KDM4C	Breast, lung, prostate, melanoma, lymphoma
	NO66	Renal cell, colorectal
Histone mutation	H3K36M/I	Glioblastoma, head and neck squamous cell carcinoma, chondroblastomas, sarcoma
	H3G34V/R/D	Glioblastoma, glioma, chondroblastomas, sarcoma, colon
Other factors	ASF1	NA
	CTK1	NA
	IDH1	Acute myeloid leukemia, glioma
	SPT6	Skin, bladder, colorectal

**Fig. 2:**
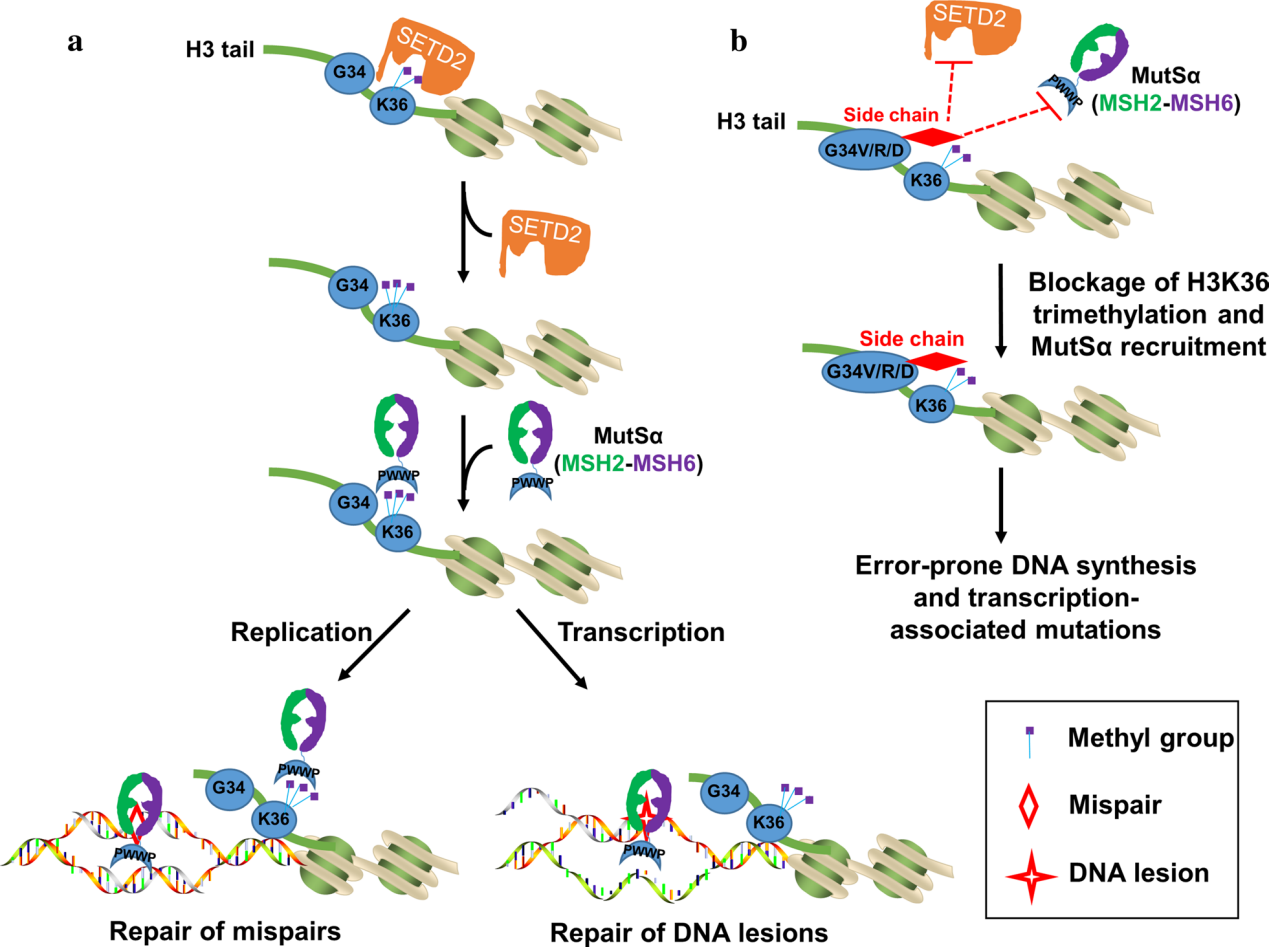
Impact of histone modifications and mutations on MMR. **a** Under normal circumstances, SETD2 interacts with and trimethylates H3K36. The resulting H3K36me3 recruits MutSα to chromatin by interacting with the PWWP domain in the MSH6 subunit of MutSα. The chromatin-associated MutSα then recognizes mismatches or DNA lesions and triggers downstream MMR reactions to correct the mispairs generated during DNA replication (left) and to remove DNA lesions in the transcribed strand during transcription (right). **b** When H3G34 is mutated into R, V, or D, the large side chain in these residues creates steric clashes with the cavity of the SETD2 catalytic domain, preventing H3K36 from being trimethylated. In addition, the large side chain also blocks the H3K36me3-MutSα interaction, even if H3K36me3 is available. In both cases, MutSα is not recruited to chromatin, leading to error-prone DNA synthesis and transcription-associated mutations

Our group recently found that H3G34V/R mutations also block H3K36 methylation by methyltransferases and result in MMR deficiency [16]. This is because these mutations inhibit H3K36′s interactions with SETD2 and MutSα. Thus, cells carrying these mutations display MMR-deficient phenotypes, including microsatellite instability (MSI) and elevated mutation frequency at the *HPRT* locus (Fig. 2). The co-crystal structure revealed that the SET domain of the SETD2 protein adopts a closed conformation for the H3K36 peptide [15], and G34V/R mutations significantly increase the space blockage resulting from the big side chain of V/R residues, which impairs H3K36 methylation [16]. Similarly, Voon et al. [36] revealed that G34V/R mutations inhibit H3K36 demethylase KDM4 to modulate the H3K36me3 pattern in pediatric glioblastoma. Therefore, both K36M/I and G34V/R mutations inhibit H3K36 methylation and are promising biomarkers for MMR-deficient cancers.

It is worth noting that these histone mutations frequently target the H3.3 variant but not the H3.1 or H3.2 variants in GBM and gliomas [13, 14, 37]. Glioma cells are terminally-differentiated cells with limited cell division. More importantly, H3.3 are preferentially incorporated into nucleosomes during transcription by H3.3-specific chaperons [38]. The question is how these mutations could target MMR, which is commonly regarded as a process coupled to DNA replication in dividing cells. One possible answer is that H3K36me3-mediated MMR also functions during transcription [17, 21]. Thus, it is feasible that H3.3K36me3 is important for MMR to maintain genome stability during transcription in the non-dividing neuron cells (Fig. 2a). Therefore, these findings largely broaden what the histone code regulates in MMR and tumorigenesis.

### Chromatin remodeling in MMR-mediated cytotoxic response

In addition to repairing mismatched DNA, MMR also functions in non-canonical ways to promote cytotoxic responses to cell stresses, such as *O*^6^-methylguanine (*O*^6^-mG)- and oxidation-induced cell apoptosis [4, 39]. MMR proteins recognize and incise the *O*^6^-mG-T mispair, but cannot correct it, which leads to a futile repair cycle and, finally, apoptosis. Similar to its role in replication-coupled repair, CAF-1 also suppresses MMR activity in response to DNA methylating drugs by packaging DNA that contains *O*^6^-mG-T mispairs into nucleosomes to prevent it from degrading [6], which may lead to cell resistance to methylating agents, such as methyl-nitro-nitrosoguanidine (MNNG). Similarly, Smarcad1 may sensitize cells to methylating agents by promoting MMR-mediated cytotoxic responses. Since MMR is important for cancer cell killing mediated by methylating drugs, and since chromatin regulation of MMR is important in vivo, future studies are required to investigate the detailed functions of chromatin remodeling factors in MMR and their potential as targets for sensitizing cancer cells to methylating drugs.

### Impact of histone methyltransferases and demethylases on H3K36me3 and MMR

It is well-known that MMR deficiency causes cancers characterized by MSI [2, 4, 40]. However, not all MSI-positive cancers display MMR gene defects [41, 42]. The underlying mechanism for these MSI cancers was not clear until H3K36me3 was identified as an essential regulator of MMR in vivo [5]. Thus, defects in H3K36me3 are considered a promising biomarker for MSI-positive cancers. Recent studies have shown that MMR deficiency or MSI benefits from immune checkpoint inhibitor therapy for patients with many types of cancer [43, 44]. Thus, a comprehensive list of factors that influence H3K36me3 levels should be examined before considering patients with MSI-positive tumors for immunotherapy.

SETD2 is the major methyltransferase that converts H3K36me2 to H3K36me3 in mammalian cells [10]. However, H3K36me3 metabolism is tightly regulated by multiple histone methyltransferases, demethylases and other factors. These methyltransferases include the SET domain-containing proteins and the DOT1-like proteins, i.e., SETD2, SETD3, SETMAR, NSD1, NSD2, NSD3, ASH1L, and SMYD2 [10, 45]. Recently, another methyltransferase, PRDM9, was also reported to trimethylate H3K36 in certain contexts [46]. In contrast to histone methyltransferases, histone demethylases, such as KDM2A, KDM2B, KDM4A, KDM4B, KDM4C, and NO66, can remove methyl groups from H3K36 [47]. In addition, histone chaperons or deposition factors are also important for properly assembling different H3 variants into nucleosomes and establishing corresponding methylation patterns [48, 49]. As an important epigenetic regulator, H3K36 methylation modulates many cellular/chromatin functions, and defects in H3K36me3 metabolism are susceptible to causing human diseases [45, 47], including cancers.

Frequent mutations in the aforementioned methyltransferase genes have been identified in different cancer types (Table 1). For example, the SETD2 loss-of-function mutations were found in renal carcinoma [50, 51], lung cancer [52, 53], gastrointestinal cancer [54], and hematologic malignancies [55, 56], indicating that SETD2 is a tumor suppressor. Similarly, other methyltransferases have also been reported susceptible to causing different types of cancer (Table 1). However, the outcome of these mutations targeting histone writer enzymes, especially the NSD family, is complex, as they lead either to losses of function or to gains of function [10]. Since H3K36me3 is essential for MMR in vivo, H3K36me3 depletion resulting from the loss of H3K36 mono-, di- and tri-methyltransferases will result in MMR deficiency and genome instability. It is also possible that these enzymes interact directly with MMR proteins to regulate MMR function in vivo. Therefore, further studies are required to demonstrate whether and how mutations of these histone methyltransferases impair H3K36 metabolism and/or MMR to promote cancer development.

In addition to histone methyltransferase mutations, depleting H3K36me3 by overexpressing H3K36me2/3 demethylases, e.g., KDM4A-C, also disrupts MSH6 chromatin localization and induces MMR-deficient phenotypes [57]. Consistent with the idea that histone demethylases function against SETD2 in MMR, mutation-led upregulation of these histone demethylases, resulting from gene fusion, amplification or overactivation [58–60], is also susceptible to causing various types of cancer (Table 1). It is likely that overexpression of these genes erases the level of H3K36 methylation in cells, which disrupts the H3K36me3-mediated MMR pathway and causes genome instability. Thus, these genes are also promising biomarkers for H3K36me3-MMR deficient cancers.

### Other H3K36 methylation regulators

Besides histone methyltransferases/demethylases and histone H3 mutations, other factors are also involved in regulating normal H3K36 methylation patterns in cells (Table 1). For example, IDH1 is frequently mutated in gliomas, and its metabolic product 2-hydroxyglutarate inhibits lysine demethylase activity [13, 61]. The histone chaperons ASF1, CTK1 and SPT6 have been demonstrated to modulate transcription-associated SETD2-mediated H3K36 methylation [48, 49]. Thus, these factors may also modulate H3K36me3-mediated MMR in vivo. Future studies are required to understand the mechanisms by which these factors regulate MMR, which will provide a more accurate and complete understanding of non-classical mechanisms by which abnormal epigenetic factors cause MMR defects and cancer susceptibility, as well as new targets or strategies for preventing and treating tumors.

## Conclusion

MMR is a replication-coupled reaction. Even though the reaction has been reconstituted [62–64], it is not yet fully understood how MMR and DNA replication are coordinated at the replication fork and how chromatin structures modulate the coupled MMR and replication processes. Identifying the involvement of histone mark H3K36me3 and chromatin remodeling factors in regulating MMR in vivo only marks the beginning of this exciting new area. We believe that future studies will identify other histone modifications and/or chromatin factors, in addition to H3K36me3 and the reported chromatin remodelers, that regulate the MMR system. Given the importance of MMR in cancer etiology and therapy [2, 41, 65], it is expected that these new factors will greatly advance cancer diagnosis and treatment.

## Data Availability

Not applicable.
