# Correction to: DNA mismatch repair in the context of chromatin

**DOI:** 10.1186/s13578-020-00447-7

**Published:** 2020-07-02

**Authors:** Yaping Huang, Guo-Min Li

**Affiliations:** Department of Radiation Oncology, University of Texas Southwestern Medical Center, Dallas, TX 75390 USA

## Correction to: Cell Biosci (2020) 10:10 10.1186/s13578-020-0379-7

In the original publication of this article [1], one of the supporting sources was missing in the Acknowledgements part. The correct Acknowledgements appear below:
